# Containment strategy during the COVID-19 pandemic among three Asian low and middle-income countries

**DOI:** 10.7189/jogh.12.05016

**Published:** 2022-05-21

**Authors:** Jun Jiao, Leiyu Shi, Haiqian Chen, Xiaohan Wang, Manfei Yang, Junyan Yang, Meiheng Liu, Xianchun Yi, Gang Sun

**Affiliations:** 1 Department of Health Management, School of Health Management, Southern Medical University, Guangzhou, Guangdong, China; 2 Department of Health Policy and Management, Bloomberg School of Public Health, Johns Hopkins University, Baltimore, Maryland, USA; 3 Yichun Hospital of Traditional Chinese Medicine, Yichun, Jiangxi, China

## Abstract

**Background:**

COVID-19 has not been effectively controlled, seriously threatening people’s health and socioeconomic development. This study aims to summarise the successful experiences and lessons in containment strategy learned from Asian Low- and Middle-Income Countries (LMICs) during the COVID-19 pandemic and analyse the effectiveness of their measures to provide lessons for LMICs in general.

**Methods:**

This is a retrospective study on the effectiveness of China, India, and Vietnam’s containment strategies. The objective was to assess the effectiveness of measures taken for COVID-19 and provide lessons for wider LMICs in controlling and preventing the COVID-19 pandemic.

**Results:**

As of June 16, 2021, the Indian epidemic was in the declining part of the rebound stage, with a total of 21 521.900 cases per million and 276.740 deaths per million – both the highest among the three countries. Entering the normalised prevention and control stage, China stably remained at a total of 63. 615 cases per million and 3.211 deaths per million. Vietnam's number of new cases per million was very low in the first stage and almost stagnant except for cluster epidemics. In May 2021, the number of new cases per million started to rapidly increase, but the total of deaths per million was at the low level of 0.627.

**Conclusions:**

A high attention to epidemics at early stages, strict border control measures, and synchronization of government and population on COVID-19 prevention and control opinions and behaviours play important roles in designing containment strategies. In addition, rapid close contact tracing and large-scale nucleic testing are good options for response to cluster epidemics.

As of June 16, 2021, there were 176 156 662 confirmed cases and 38 154 486 deaths of COVID-19 globally. Even though a year and a half passed since the first reported cases, the global pandemic has not been effectively controlled, with large-scale outbreaks and cluster epidemics still emerging. On February 24, the World Health Organization (WHO) published the COVID-19 Strategic Preparedness and Response Plan, which builds on what we have learned about COVID-19 and our responses while focusing on new challenges [1]. Vaccines are considered an effective means of controlling COVID-19, but vaccination rates in Asian Low- and Middle-Income Countries (LMICs) are generally low, with 35.89 doses per 100 people in India, 8.28 in Vietnam, and 8.812 in Bangladesh, due to limitations of national technologies and economic development [2]. While scaling up vaccination, non-pharmaceutical social prevention and control measures remain priorities for Asian LMICs in fighting COVID-19.

Asian countries such as China, Thailand, Japan, Korea, and Vietnam are early reporters of COVID-19 cases, and their responses have provided experience and models for other countries. Asian countries mainly adopt two response strategies, the first being mitigation measures, such as those adopted by Japan and Singapore. For example, Japan recommends home isolation for mildly ill patients, and Singapore focuses on a graded diagnosis and maintaining normal daily life [3]. The second response strategy consists of containment measures akin to those adopted by China, Vietnam, and South Korea, including blocking policies, limiting social distance, and actively tracking and managing close contacts [4,5].

In this paper, three Asian LMICs – China, India, and Vietnam –with a 2020 Gross national income (GNI) below US$12 695 are selected. China has the largest population in the world at 1.4 billion and the third-largest land area. As the world's second-largest economy, close international exchanges have brought great challenges to China's epidemic prevention and control. However, China effectively and rapidly controlled the epidemic and stably maintained it in the normalised prevention and control stage. India's population structure is young, with only 6.5% of the population over 65 years old [6]. It faces many issues, such as poor living conditions, overcrowding, lack of hygiene and sanitation [7]. However, India delayed the epidemic peaking in early stages through a five-stage lockdown. Vietnam, a communist country on the southeast Asian coast, is the world's third-largest rice exporter. Vietnam implemented its social health insurance program in 1992 [8], and has been used as a model for COVID-19 response [4], achieving no change or a slight increase in cases over a long period. However, the number of confirmed cases in India and Vietnam rose drastically in 2021, and there were several cluster epidemics in China and Vietnam. This study summarises the intervention measures in Asian LMICs during large-scale outbreaks and cluster epidemics of COVID-19 and analyses the effectiveness of their containment strategies to provide lessons for wider LMICs in controlling and preventing the COVID-19 pandemic.

## METHODS

This was a retrospective study on the effectiveness of China, India, and Vietnam’s containment strategies, ie, the three countries' measures taken for containing COVID-19, based on COVID-19 data from the day of the first reported case in each country to June 16, 2021. This study also adopted a case study approach in analysing cluster epidemics. By assessing the effectiveness of COVID-19 containment measures, we wanted to provide lessons for wider LMICs in controlling and preventing the COVID-19 pandemic.

### Survey and statistics of COVID-19 data

This paper investigated new cases per million and total deaths from the day of the first reported case of COVID-19 in China, India, and Vietnam to June 16, 2021, including cases in cluster epidemics. The inclusion criteria for confirmed cases of COVID-19 in China is based on the *Chinese Clinical Guidance for COVID-19 Pneumonia Diagnosis and Treatment* published by the Chinese Health Commission. Classification of clinical manifestation was added in Hubei Province in February 2020, and it included suspected cases with imaging features of pneumonia to clinical diagnosis cases. Data on COVID-19 cases are derived from the Coronavirus Resource Centre at Johns Hopkins University (https://coronavirus.jhu.edu/?from=groupmessage). China’s data before November 22, 2020, are supplemented by daily outbreak notifications issued by the Chinese Health Commission (http://www.nhc.gov.cn/xcs/yqtb/list_gzbd.shtml) (see Figure S1 in the **Online Supplementary Document**).

### Retrieval and statistics of interventions

Interventions were searched from the first reported case of COVID-19 in China, India, and Vietnam to June 16, 2021. The PubMed, Web of Science, and Scopus bibliographic databases were searched for hotspot policy using the following search string: ((SARS-CoV-2) OR (COVID-19)) AND ((policy) OR (response) OR (strategy) AND ((China) OR (India) OR (Vietnam))). This search strategy extracted “public health”, “face masks”, “lockdown/city closure”, and “isolation/quarantine” as key policies. Since COVID-19 is a public health crisis, these terms are representative of non-pharmaceutical interventions recommended by the WHO, and widely used for COVID-19 response. In addition, interventions in official websites were collected, such as China's Health Committee (http://www.nhc.gov.cn), Ministry of Health of Vietnam (https://moh.gov.vn/), and Ministry of Health and Family Welfare of India (https://www.mohfw.gov.in/) (see Appendix S1 in the **Online Supplementary Document**).

After collecting data and interventions, we mapped national outbreak curves over time, sorted through the policies, and listed governance and socioeconomic measures, movement restrictions, border control measures, and public health measures, while also separately listing cluster epidemic response measures.

## RESULTS

### National measures to prevent and control COVID-19

Vietnam and India border China, with whom they have close economic and cultural exchanges. China reported initial COVID-19 cases in December 2019, and India and Vietnam both reported their first domestic cases of COVID-19 in January 2020. Initially, in the absence of knowledge of the aetiology, physiology, and epidemiology of the coronavirus, China quickly adopted non-pharmaceutical interventions for social prevention and control. Typical measures included lockdowns, limiting social distance, suspension of schools, isolation and self-isolation, face masks, frequent hand washing, increasing hospital beds, and coordinating medical staff. In the normalized prevention and control stage, the focus of prevention and control shifted to border control measures. China also used big data of trip records and health codes. In cluster epidemics, China focused on epidemiological investigation, precise prevention and control, and strict management to accurately identify close contacts and delineate the scope of prevention and control.

Similar containment measures have been taken in India and Vietnam. Before the country’s first COVID-19 case, border prevention and control measures, such as flight and visa suspensions and entry and exit restrictions were already in place. The typical intervention policy in India was a five-stage lockdown to restrict movement. Regionalized management of cluster epidemics is adopted to investigate cases and control the region. Vietnam had a strict policy of country-level lockdown and had developed grassroots cross-sectoral cooperation and established the “3 in advance” (identify, proactively prevent, and plan) and “4 on the spot” (onsite resources, onsite leadership, onsite facilities, and onsite logistics) strategies as the principles of management. For cluster epidemics, strict public health measures were taken. **Table 1** summarizes the major measures taken for COVID-19 in China, India, and Vietnam, including governance and socioeconomic measures, movement restrictions, border control measures, and public health measures. **Table 2** separately lists cluster epidemic response measures.

**Table 1:** The major measures taken for COVID-19 in China, India, and Vietnam

Country/category	Measure	China	India	Vietnam
Governance and socioeconomic measures	State of emergency declared	1.Provinces steadily activated level I emergency response.	1.COVID-19 was declared a “notified disaster” under the Disaster Management Act.	1.Moved up alert levels.
		2. On May 7, 2020, entered normal prevention and control stage.		
	Emergency administrative structures activated or established	1. Established an epidemic response and disposal leadership team.		1.Established COVID-19 Working Group host by Deputy Prime Minister [4].
		2. A strong epidemic command system ensures the efficiency of decision-making and synchronization of policies.		2.The district health centers and local authorities set up “rapid action teams” [9,10^]^.
	Limit product imports/exports		1. Restrict export of vaccine	1.Government held back signing new rice export contracts.
	Economic measures	1. Free treatment to COVID-19 cases [11]. 2. Promoted a decentralized economy and issued consumption coupons.	1. Announced a 1.7 trillion-rupee economic relief program.	1.Around VND7630 billion was financed for free and unemployed laborers.
			2. Released a stimulus package of about Rs730 billion.	
	Social policies	1. The infected should be treated in designated hospitals by doctors and with all necessary resources guaranteed.	1. For violation of lockdown policies, should be taking action under relevant laws.	1.A risk assessment for 2019-nCoV was conducted [12].
		2. The principle of early detection, reporting, quarantine, and treatment be strictly observed.		2.The principle of “3 in advance” and “4 on the spot” was implemented [13].
				3. Issued National Response Plan and guidelines of diagnosis and treatment [14].
Movement restrictions	lockdown	1. On January 23, 2020, Wuhan lockdown.	1. A five-stage lockdown was implemented.	1.Imposed a national lockdown from April 1 to 15, 2020 [15].
	Schools closure	1. Announced an extension of the opening of schools around the country.	1. New Delhi announced all elementary schools closed for three weeks.	1. All schools were closed from the lunar New Year holiday [16].
	Social distancing	1. Wuhan strict closed traffic control.	1. Rails shut down and interstate buses and the metro were suspended.	1. Reduce domestic flights.
		2. Set up one-meter lines in public places.		
	Limit public gatherings	1. Closed entertainment venues; encouraged people to telecommute from home and businesses.	1.Proposed to adopt a “work from home” policy.	1. Banned, suspended, or reduced traditional holidays [4]. Mitigated bar and outside activities [17].
			2. Mass celebrations were banned.	2. Social distance was required nationwide.
		2. Implementation of a closed community, grid-based management.	3. Called all Indians to stay out from 7:00 am to 9:00 pm on Mar 22, 2020.	
Border control measures	Visa restrictions	1.Suspend the entry of foreigners with valid Chinese visas and residence permits.	1. All visas for foreigners were temporarily invalidated.	1. Entry was suspended for all foreigners.
	International flights suspension		1. All international flights were banned. And from Jul 17, 2020, reopened some routes.	1. Ban to all international travel and Cancellation of all foreign flights [15].
				2. Cancellation of all foreign flights
	Border closure	1. Temporarily closed seven border stations.	1. On Mar 11, 2020, announced border closures.	1.On January 28, 2020, closed borders with China [15].
		2. Implemented a movement against illegally crossing the nation's borders.	2. Issued a travel warning asking to avoid non-essential travel to China.	2. The travel restriction policy extended to countries affected by COVID-19.
	Health screenings and border crossings	1. Strengthened health quarantine at ports and epidemiological history checks.	1. All travellers should submit a self-declaration form on the online portal.	1. Compulsory health declarations at all international ports.
		2. Implemented the health declaration system for entry and exit personnel.		
	Immigration management	1. Adopted 14-d centralized quarantine for inbound travellers.	1. 14-d mandatory quarantine was imposed on travellers from severe outbreaks countries.	1. Incoming travellers shall be subject to centralized quarantine for 14 d.
Public health measures	Isolation and quarantine policies	1. Close contacts or infection risks after epidemiological investigation will be taken to local sites for a 14-d quarantine.	1. Construction of Medical Center, Cabin Hospital, conversion trains for temporary square cabin hospitals, and isolation wards.	1. Organized quarantine quarters.
				2. Classified isolation to three levels.
		2. Building more makeshift hospitals.		3. Passengers suspected shall be kept isolated timely.
	vaccination	1. Launch vaccination of key populations.	1. Launched vaccination campaign.	1.Issued vaccination schedule.
	Surveillance and monitoring	1. Use big data to conduct epidemiological surveys and map out the epidemic.		1. Promoted electronic medical statement and contact tracing apps.
		2. Adopted active close contact tracing.		2. Used contact tracing apps and medical students to the fourth level of contact.
	wear protective gear in public	1. Strictly enforced the wearing of masks outside.	1. Some states introduced mandatory rules: all travellers must wear masks.	1. Promoted the use of masks in public places.
	Other public health measures enforced	1. Launched health code system [18].	1. Launched mobile app for registering for home quarantine, checking bed availability, and requesting ambulances.	1. The rapid development of diagnostic test kits.
		2. Vigorously carry out patriotic health campaigns.	2. Launched a public education campaign to prevent the virus.	2. Information related to COVID-19 was released to the public through all media platforms.
		3. Established an epidemic information release mechanism.		

**Table 2:** Cluster epidemic response measures taken for COVID-19 in China, India, and Vietnam

**Cluster epidemic response measures**	1. In June 2020, COVID-19 outbreak in Beijing's Xinfadi market. 1) Quickly located the source, and immediately took measures to close the market and fully seal it, prohibiting people and goods from entering or leaving the market. 2) Took closure and control measures for the surrounding neighbourhoods. 3) Implemented hierarchical classification and precise prevention and control, strict management of key places and key industries. 4) Conducted extended range of nucleic acid testing. 5) Required people related to medium and high-risk streets and towns and Xinfadi Market to be prohibited from leaving Beijing. 6) Banned open domestic cross-provincial and municipal group travel business to reduce crowd mobility.	1. On March 24, 2021, a double mutation of coronavirus was discovered in Maharashtra. 1) On March 28, the state began a “lockdown-like” universal curfew, which was in effect from 20:00 to 7:00 daily from Monday to Friday; all day on Saturday and Sunday; and no gatherings of more than five people were allowed. 2) Restaurants are not allowed to provide in-store dining services, only take-out is allowed; cinemas, theatres, and other public events are closed, and then extended until Jun 15. 3) Persons entering the state from other parts of India by any mode of transportation are required to carry a negative nucleic acid test [19]. (4) Drones are used to monitor physical distances and apply a cluster containment strategy: If three or more patients are diagnosed, all houses within a 3-km radius are surveyed to detect additional cases and track contacts [20].	1. The epidemic is increasingly spreading in major cities such as Hanoi and HCMC. 1) A ban on assemblies of 20 or more people indoors and 10 or more people in public places was issued in late March 2020. 2) Recreational, cultural, and sporting activities in public places were banned. 3) non-essential businesses, including restaurants, bars, beauty salons, barber stores, hair salons, massage parlours, spas, and gyms, were closed with a recommended physical distance of 2 m.
	2.A cluster epidemic occurred in Guangzhou in May 2021. 1) On May 26, Liwan District organized the first full nucleic acid testing, and later organized several rounds of district-wide and city-wide nucleic acid testing; 2) From May 29, Guangzhou implemented the classification of prevention and control, all people in some areas of Liwan District mainly at home, stop all activities that are not essential to daily life, except for a daily limit of one person per household to go out to buy the necessities of life, all other people are not allowed to go out. 3) advocate “non-essential, not out of Guangzhou, not out of the province”. on May 30, announced who leave Guangzhou need the “health code” green code and 72-h nucleic acid test negative certificate.		2.In late July 2020, following reports of an outbreak in Da Nang. 1) Authorities urgently evacuated nearly 80 000 foreigners at Da Nang airport and restarted community lockdown and social quarantine measures. 2) Residents were not allowed to go outside unless necessary, and cross-province passenger services were temporarily suspended. Festivals, religious, and other large-scale gatherings were all called off, and entertainment venues such as bars and discos were temporarily closed. 3) Businesses, schools, and sports stadiums are allowed to remain open, but strict hygiene and epidemic prevention standards need to be followed in the relevant facilities. 4) To force people to wear masks in public, the government has imposed fines of VND1-3 million since Nov for those who do not comply [21].

### COVID-19 trends

**Figure 1:**
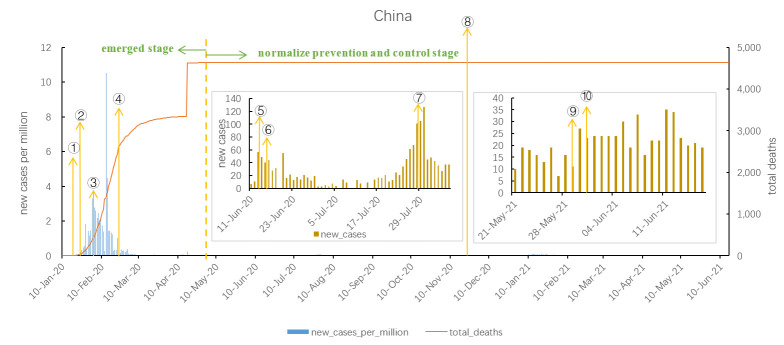
COVID-19 outbreak curve and major measures in China. 1. On January 23, 2020, lockdown Wuhan city. 2. On January 26, delay of school opening. 3. On February 7, Hangzhou launch the health code. 4. On February 25, strengthen entry-exit epidemic prevention. 5. On June 13, close the Xinfadi Market and carry out nucleic acid tests. 6. On June 16, Beijing implemented community closed management, and the relevant personnel are banned from leaving Beijing. 7. On July 28, the Xinfadi Market resumed. 8. In February, launched key groups vaccination. 9. On May 29, 2021, Guangzhou implemented the prevention and control measures based on different levels, and people in Liwan District mainly stayed at home. 10. On May 30, Passengers leaving Guangzhou are required to present green code of Health code and negative nucleic acid test within 72 hours.

As shown in **Figure 1**, the COVID-19 epidemic in China is divided into two stages: the emerged stage and the normalized prevention and control stage, which was cut off on May 8, 2020. During the emerged stage, the initial number of new cases was low, and from late January to early March 2020, China experienced the first wave of the COVID-19 outbreak, which peaked in February with a similarly sharp increase in the total number of deaths; after March 7, the daily number of new cases fell below 100, and remained basically zero except for cases imported from abroad. In the normalized prevention and control stage, sporadic cases and cluster epidemics are intertwined. But from April 7, 2020, to June 16, 2021, the total number of deaths increased by only 4 cases. And as of June 16, 2021, the total number of cases was 63.615, and total number of deaths per million was stable at 3.221.

**Figure 2:**
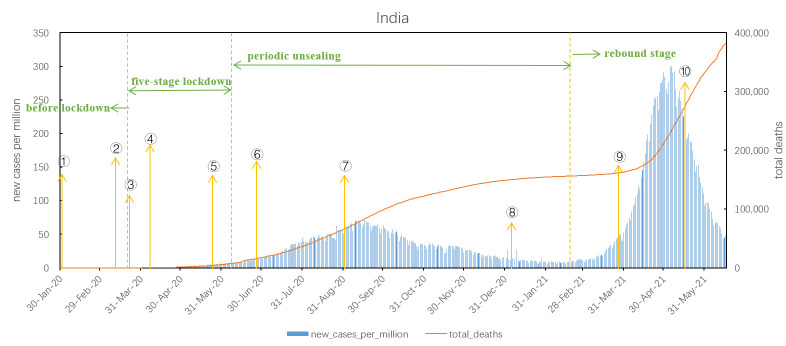
COVID-19 outbreak curve and major measures in India. 1. On January 31, 2020, the Directorate General of External Trade issued a ban on the export of personal protective equipment. 2. On March 11, India announced border closure and temporary suspension of tourist visas. 3. On March 22, flights were restricted, and local traffic was suspended. 4. On April 9, masks became mandatory. 5. On May 25, domestic flights resumed. 6. On Jun 27, a mobile cabin hospital was built at the Bangalore International Exhibition Centre. 7. On September 1, workplaces, public transport, restaurants, gyms, and more reopened. 8. On January 6, 2021, a nationwide COVID-19 vaccination campaign was launched. 9. On March 28, a total curfew was imposed. 10. On May 16, new Guidance on COVID-19 containment (rural) was issued.

As shown in **Figure 2****,** the epidemic in India is divided into four stages according to its major measures taken for COVID-19: 1) pre-lockdown stage, 2) five-stage lockdown, 3) periodic unsealing, and 4) rebound stage. Before lockdown, the number of new cases was low, under 50. From May 25 to June 30, 2020, India began a five-stage lockdown with a low growth rate of new cases per million. From June 8, 2020, India entered the periodic unsealing stage, with new cases per million continuing to increase based on the five-stage lockdown and reaching the peak of first wave in mid-Sep, with the total number of deaths reaching 90 000. After the second epidemic wave in mid-February 2021, India entered the rebound stage, which peaked in early May. The total number of deaths per million reached 276.74 as of June 16, 2021.

**Figure 3:**
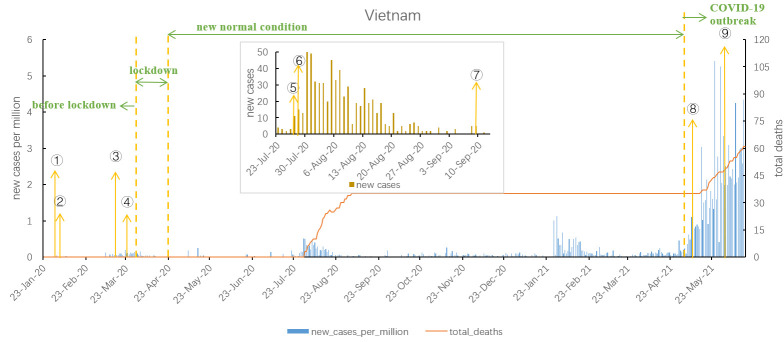
COVID-19 outbreak curve and major measures in Vietnam. 1. On January 31, 2020, the Prime Minister issued that banning, suspending, or narrowing traditional festivals. 2. On February 5, COVID-19 information and prevention measures were released to the public through media platforms. 3. On March 16, wearing masks was mandatory in public. 4. On March 25, all international travellers were banned. 5. On July 27, Da Nang City was lockdown. 6. On July 28, a large-scale nucleic acid testing was carried out. 7. On September 11, Da Nang City was lifted. 8. On May 7, 2021, a COVID-19 vaccination schedule was issued. 9. On May 31, Ho Chi Minh City introduced a 15-day social distancing measure.

As shown in **Figure 3**, the epidemic in Vietnam is divided into four stages: 1) pre-lockdown stage, 2) lockdown stage, 3) new normal condition stage, and 4) COVID-19 outbreak. On January 23, 2020, the first case of COVID-19 was reported in Vietnam. During the pre-lockdown stage, there was no increase in new cases, but after the discovery of COVID-19 cases on an international flight from London to Hanoi in March 2020, the second wave emerged and the number of new cases per million increased slightly. Vietnam rapidly entered the lockdown stage, after which the epidemic was stable. On April 24, 2020, Vietnam issued a directive on the prevention and control strategy under the “new normal condition”. There were several small-scale outbreaks during the new normal condition. In May 2021, when the number of new cases per million came over 1 and total deaths also increased, Vietnam entered the COVID-19 outbreak stage. As of June 16, 2021, the total number of cases per million reached 121.165, and total number of deaths per million was 0.627.

**Figure 4:**
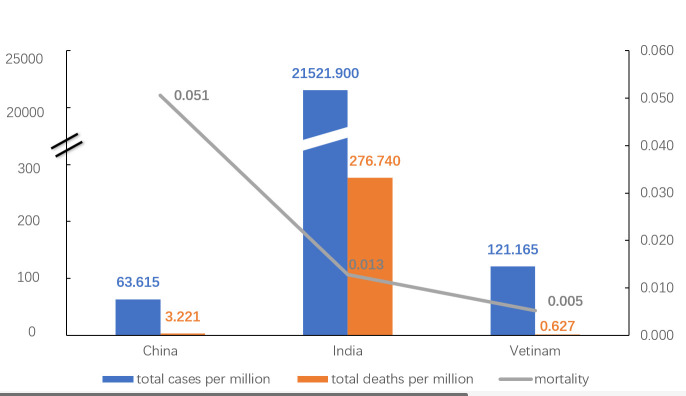
Total number of cases per million, total number of deaths per million and mortality of COVID-19 in China, India, and Vietnam.

As shown in **Figure 4**, India had a higher total number of cases per million and total number of deaths per million than China and Vietnam, but a lower mortality rate. **Figure 1**, **Figure 2**, **Figure 3** and **Figure 4** show different dynamics of COVID-19 in the three countries, with the first wave in China occurring early and lasting for a short period, followed by several clusters’ epidemics but no large-scale outbreaks. There were two waves in India. Compared with the first wave, the second wave showed an upsurge of new cases and a shorter epidemic cycle, with aggregated outbreaks hidden in the explosive growth of cases. In Vietnam, epidemic outbreaks occurred in the early stages, and the large-scale outbreak occurred in April 2021, later than in other countries.

## DISCUSSION

This study describes major measures adopted by three Asian LMICs, China, India, and Vietnam, in response to COVID-19 and their effectiveness. The three Asian LMICs implemented containment strategies such as lockdowns, limiting social distance, and epidemiological survey to interrupt epidemic spread. Currently, many researchers have demonstrated the effectiveness of social interventions such as lockdown, facial masks, and limiting social distance by using epidemiological and transmission theories and constructing simulation models [22,23]. We will discuss the successful experiences and shortcomings of three countries in responding to COVID-19 in terms of governance and socioeconomic measures, movement restrictions, border control measures, public health measures, and cluster epidemic response strategies. Notably, differences in sociocultural environments, political structures, economics, and sanitary condition between the three countries and other countries may pose a challenge to the applicability of the measures reported.

### Governance and socioeconomic measures

Governance and socioeconomic measures are the guiding strategies in the prevention and control strategy of COVID-19. The declaration of an emergency and the activation or establishment of emergency administrative structures symbolise that a country is taking a pandemic seriously. Vietnam conducted a national risk assessment on January 20, 2020, developed a response plan and treatment guidelines [21], and established the COVID-19 outbreak task force quickly after the first case [4], which was key to successfully controlling COVID-19 in Vietnam [14]. China established and improved the “three systems of one case” emergency management system since the 2003 SARS outbreak, but the link between hospitals and primary health care organisations was weak [24], resulting in a delayed response to COVID-19. China worked quickly after confirming a case of pneumonia of unknown origin. India did not pay much attention to the first COVID-19 case and only declared it a disaster when the number of cases started to grow. In the early stages of COVID-19, there was less information on transmission and severity, and some countries did not pay sufficient attention to it, which contributed to the global pandemic [25]. High attention from national to local level in early stages, advanced preparation, rapid policy response, and improvement of emergency management system were the key factors in successfully preventing and controlling COVID-19 [26].

Having faced an increase in domestic cases, both China and India took steps to provide more access to medical care by renovating medical centres and building square-cabin hospitals. Timely treatment is an effective way to isolate infection sources and reduce mortality. China mobilized national medical resources to support Hubei and provided free treatment to confirmed patients, which improved admission and cure rate of patients; Vietnam gathered local health workers and medical students to carry out rescue and epidemiological investigation, rather than only relying on specialized work units, to avoid shortage of medical human resources. Additionally, India and Vietnam each forbid exports of certain medical supplies and food to ensure domestic supplies were adequate.

In response to the economic downturn, all three LMICs adopted economic policies such as lowering tax rates, safeguarding employment, and carrying out economic stimuli. However, the epidemic led to a sharp increase in unemployment in India and Vietnam, and simple economic policies could not properly supplement the residents' livelihood. Vietnam still insisted on social restriction measures and imposed state control on resumption of work and production; India implemented economic policies on resumption of work and production when the first wave was not under control, which is an important reason for the gap between Vietnam and India.

### Movement restrictions

Movement restrictions have played an important role in limiting the spread of local outbreaks, which are the most widely used and most stringent type of social intervention in containment strategies. The three countries implemented lockdown early on. China implemented a 76-day city lockdown on Wuhan, India implemented a five-stage lockdown, and Vietnam extended lockdown several times. A lockdown in early stages of epidemic played a key role in interrupting the spread of virus, flattening the growth curve of epidemic, and preparing medical resources [27]. Compared to high-income countries, Asian LMICs were abler to carry out large-scale social interventions, such as early-stage lockdowns. There are multiple reasons behind this phenomenon. First, cultural characteristics of Asian LMICs influence citizens to be more cooperative. The three countries or Asian LMICs consist of many nations with tight cultures, which are more using positive coping behaviours, and their citizens are more willing to cooperate under threat [28,29]. Second, public risk perception affects public support for different strategies. Cultural traditions of Asian LMICs are relatively conservative and introverted, and people are more willing to avoid risks, opting for containment rather than mitigation strategies [30]. As for the mainstream values of society, Asian LMICs, especially socialist countries, pay more attention to collectivism and cohesion [31]. However, the harmful effects of prolonged lockdown on economic development are self-evident and have a greater impact on LMICs. China had a Gross Domestic Product (GDP) growth of 2.3%, Vietnam of 2.9%, while India had a decline of -8% in 2020 [32]. 85% of workers in urban India are informal workers, most of whom are domestic workers, street vendors, and scavengers [33], and lockdown has negatively impacted their sources of income and increased hunger and food deprivation. In addition, the staged resumption of work and production policy, which emphasized social distances and reduced employee movement restrictions, posed challenges to production reorganization [34]. However, India failed to respond well and relaxed measures prematurely when there were still large sources of infection in community and low vaccination rates, thus leading to the second wave. In contrast, China and Vietnam emphasized the importance of movement restrictions in the “new normal” stage and combined them with public health measures to maintain the stability the number of cases while slowly implementing the resumption of work and production and the orderly return of students to school.

### Border control measures

When COVID-19 became a global pandemic, border control measures, which are the first barrier to virus entry, became important. Their stringency determines the impact of imported cases on the domestic epidemic. In many countries, the first cases of COVID-19 were transmitted through international flights, but airport checks have a limited role as standalone measures [35]. Travel restrictions, visa suspensions, international flight control, and other entry restriction measures are the first prevention and control measures taken by many countries. India and Vietnam closed their borders with China, to block the importation of cases. However, they neglected to grasp the international outbreak dynamics, leading to large infections in India from Italian tour groups in March 2020 [25]. The main sources of foreign importation to Vietnam were the UK, France, and the US [16]. Moreover, there are partial deficiencies in health screening and isolation of immigrants in the three countries, generating secondary transmission. India quarantined international travellers coming from countries with severe outbreaks for 14 days only. The effectiveness of the quarantine policy was greatly reduced after home quarantine was allowed in June 2020. Vietnam adopted a 14-day mandatory centralized quarantine [36], but faced problems of formalism, as isolation and prevention measures were not set in place. For example, there were random outings and cross-infection in quarantine. China adopted a quarantine policy with different isolation periods for international travellers at different times, adopting a centralized 14-day quarantine in the early periods and extending the quarantine period after the spread of delta virus. However, some port cities have seen the spread of imported cases from abroad due to siting and inadequate disinfection of isolation sites, and the presence of asymptomatic infections.

### Public health measures

Public health measures are frequently used to prevent and control the transmission of COVID-19, often in combination with other measures, and are primarily people-related. Masks are considered an effective protection measure against COVID-19, which is transmitted through respiratory droplets and indirect contact [37]. Some countries resist masks and the refusal to wear a mask is used as a symbol of democratic freedom and political identity. All three countries recognised the importance of masks and promoted or enforced wearing of masks in public places, but the three countries differed in policy stringency and the extent to which the public followed the mask mandates. In the early stages of COVID-19, China ran out of masks, and local governments took various measures to gradually secure the domestic supply by making reservations online, selling them at low prices, and promoting the resumption of work and production at mask manufacturing plants. After the epidemic normalised, wearing masks was still required in public places such as transport and shopping malls. Vietnam was also an early adopter and promoter of wearing masks. Some Indian cities urged people to wear face masks. Mumbai introduced a policy of a minimum six-month imprisonment for refusing to wear a mask. However, Mumbai also faced mask shortages and problems with production capacities, because of which the wearing of homemade masks was promoted, despite the lack of evidence on their effectiveness in preventing COVID-19 transmission. As COVID-19 continues to affect the world, encouraging the public to continue to take personal protective measures and promoting vaccination is a necessary part of ensuring a normal productive life in the COVID-19 pandemic.

The press and media played a significant role in educating the public and raising awareness of and compliance with policies, drawing attention to the importance of information and implementing policies. All three countries use the media to disseminate timely information about COVID-19 and ensure that information is reliable, timely, accurate, and transparent. Jeffrey V. Lazarus' study showed that China's satisfaction with COVID-19 policy of 80.48 far exceeded the average score of 52.95 [38]; Vietnam’s citizens had an above average risk perception of COVID-19 [39]. In contrast, Indian citizens were less aware of the epidemic and many people misunderstood policy during lockdown, which led to a large population flow from urban centres to rural areas. The role of media, the rapid implementation of policies, and the synchronization of government and population thinking and behaviour to combat epidemic were key factors in successful response to COVID-19 [13]. A social system that emphasizes the unity of the collective effort and decision-making are also important.

### Cluster epidemic response measures

A “cluster epidemic” is defined as the discovery of two or more confirmed cases or asymptomatic infections in a small area (eg, a household, a site, a unit, etc.) within 14 days, and possibility of interpersonal transmission due to close contact or possibility of infection due to shared exposure [40]. The spread of imported cases from abroad, staff at isolated sites, and the non-standardized management of imported cold chains are frequent causes of cluster epidemic.

After China entered the normalized prevention and control stage, several cluster epidemics emerged. Most cluster epidemics in China occurred in port cities and high latitude areas. In this study, the Beijing Xinfadi market epidemic on June 11, 2020, and the Guangzhou epidemic on May 21, 2021, were selected for discussion. For Beijing, the virus could have been reintroduced via cold-chain transportation of contaminated items [41], and workplace and household aggregation were predominant. For Guangzhou, there is a high risk of early spread of imported cases from abroad, and a large proportion of asymptomatic infected persons and family aggregations.

In the face of cluster epidemics with abovementioned characteristics, China changed its emergency management strategy in regions where epidemics occurred, shifting from normalized to cluster epidemic prevention and control. Neither Beijing nor Guangzhou adopted lockdown policy like Wuhan. They adopted strict exit management to prevent export, scientifically and precisely classified risk levels, and adopted corresponding measures for different populations and sites to focus resources on key areas, reduce transmission, and impact on economic and social life. Second, information technology and big data were used to identify people using health codes and trip codes, and epidemiological surveys were conducted by telephone, message, and big data trip self-examination. Through rapid epidemiological investigation, Beijing locked the Xinfadi market area within 22 hours [42]. Moreover, due to the incubation period and interstitial detoxification of coronavirus, adding to the large population, a single round of nucleic acid testing cannot provide complete screening. In Beijing, 197 infected patients were found through nucleic acid screening [43] and Guangzhou conducted six rounds of large-scale nucleic acid testing. Facing pressure on medical, financial, and human resources caused by multiple rounds of mass nucleic acid testing, China has made extensive use of “5-in-1” and “10-in-1” nucleic acid testing practices, saving medical resources, accelerating testing efficiency, and improving early detection capabilities. Residents' active cooperation is also a necessary condition for successful implementation of large-scale nucleic acid testing.

Cluster epidemics in India have continued to occur, from the first cluster epidemic in Kerala on January 30, 2020, to Maharashtra in March 2021. India cluster epidemics were hidden in a whole-country epidemic with a long duration and significant spread. It is difficult to distinguish the control measures for cluster epidemic from the overall strategy. India's cluster epidemic measures are likely regional, taken for specific epidemic areas. In the Maharashtra cluster epidemic, India applied a cluster containment strategy, targeting herd immunity and adopting “blockade-like” measures. However, larger cluster epidemics in India, coupled with virus mutations that manifest numerous infections and the weak primary health care system, lack of public health personnel, and inadequate telephone penetration added to the difficulty of rapidly contract tracing [24]. In addition, the lack of rapid diagnostic test kits and inadequate capacity for large-scale nucleic acid testing made centralized isolation appear difficult [24]. Clustered containment strategies in India cannot achieve effective control in a short period.

Cluster epidemics in Vietnam mainly occurred during the first three stages, such as the Danang epidemic and the Bach Mai Hospital epidemic in Hanoi. Vietnam's strict management of cluster epidemics is why the number of new cases remained relatively stable at single-digits or zero. Normal containment measures were implemented. For example, on the first day of the Da Nang epidemic, three relevant medical institutions were quickly sealed off and 6018 suspected cases were isolated [44]. Rapid case screening and timely contact tracing are key elements in preventing cluster epidemics [45]. Vietnam developed contact tracing applications and rapid diagnostic kits, founded grassroots cross-sectoral organisations, and organised thousands of medical students to participate in epidemiological investigations to accelerate case screening and tracing. Large-scale nucleic acid testing of local population and all people returned from Da Nang were conducted [44], while strengthening nucleic acid testing of low-risk populations to actively detect cases. In containment strategy, early detection and management of close contacts is crucial in preventing transmission, which can reduce the burden of epidemiological surveys.

A limitation of our study is that the policy search and collection was unsystematic, which could result in the omission of certain policies. Another limitation is that the study did account for the effect of virus variants on measures, which present different characteristics in transmission speed, pathogenicity, and so on [46].

## CONCLUSIONS

In containment strategy, high attention to epidemics at early stages, strict border control measures, and synchronization of government and population on COVID-19 prevention and control thoughts and behaviours are the lessons from Asian LMICs experience. The finding further supports the early adoption non-pharmacological interventions for social control measures, proving they can effectively limit the spread of COVID-19. Compared to high-income countries, Asian LMICs can more easily implement large-scale social interventions such as early-stage lockdowns. In addition, rapid close contact tracing and large-scale nucleic testing are good options for response to cluster epidemics.

## Additional material


Online Supplementary Document
