# Supplementary material for: Containment strategy during the COVID-19 pandemic among three Asian low and middle-income countries
Source: J Glob Health. 2022 May 21;12:05016. doi: 10.7189/jogh.12.05016 (PMC9123341; doi:10.7189/jogh.12.05016)
Supplement: Online Supplementary Document [file jogh-12-05016-s001.pdf]

The study retrieval process

Firstly, the MeSH in PubMed was used to search the "COVID-19" subject words and confirmed as "COVID-19" and "SARS-COV-2".

Secondly, in PubMed, Web of Science, and Scopus databases, we used the following search strings to search hotspot policy.

1. In PubMed, the search string of ((SARS-CoV-2) OR (COVID-19)) AND ((policy) OR (response) OR (strategy) AND ((China) OR (India) OR (Vietnam))) was used.

2. In Web of Science, the search string of (TS=(SARS-CoV-2 OR COVID-19) AND TS=(policy OR response OR strategy) AND TS=(China OR India OR Vietnam)) was used.

3. In Scopus, the search string of (TITLE-ABS-KEY (COVID-19 OR SARS-CoV-2) AND TITLE-ABS-KEY (response OR policy OR strategy) AND TITLE-ABS-KEY (China OR India OR Vietnam)) was used.

Finally, after obtaining policies from search results, we got "public health", "face masks", "lockdown/city closure", and "isolation/quarantine" as key policies. Firstly, COVID-19 is a typical public health event. Secondly, "face masks", "lockdown/city closure", and "isolation/quarantine" are representative non-pharmaceutical interventions recommended by the WHO. Finally, these terms were widely used as COVID-19 responses.
